# Immune response to a potyvirus with exposed amino groups available for chemical conjugation

**DOI:** 10.1186/1743-422X-9-75

**Published:** 2012-03-27

**Authors:** Carlos Alberto Manuel-Cabrera, Ana Márquez-Aguirre, Hernández-Gutiérrez Rodolfo, Pablo César Ortiz-Lazareno, Gabriela Chavez-Calvillo, Mauricio Carrillo-Tripp, Laura Silva-Rosales, Abel Gutiérrez-Ortega

**Affiliations:** 1 Unidad de Biotecnología Médica y Farmacéutica, Centro de Investigación y Asistencia en Tecnología y Diseño del Estado de Jalisco, Normalistas 800, Colinas de la Normal, Guadalajara, Jalisco 44270, México; 2 División de Inmunología, Centro de Investigación Biomédica de Occidente, Instituto Mexicano del Seguro Social, Sierra Mojada 800, Independencia, Guadalajara, Jalisco 44340, México; 3 Departamento de Ingeniería Genética, Centro de Investigación y de Estudios Avanzados del Instituto Politécnico Nacional, Unidad Irapuato, km 9.6 Libramiento Norte, Carretera Irapuato-León, Irapuato, Guanajuato 36821, México; 4 Laboratorio Nacional de Genómica para la Biodiversidad, Centro de Investigación y de Estudios Avanzados del Instituto Politécnico Nacional, Unidad Irapuato, km 9.6 Libramiento Norte, Carretera Irapuato-León, Irapuato, Guanajuato 36821, México

**Keywords:** Tobacco etch virus, capsid protein, amino groups, chemical conjugation, immune response

## Abstract

**Background:**

The amino terminus of the tobacco etch virus (TEV) capsid protein is located on the external surface of infectious TEV particles, as proposed by previous studies and an *in silico *model. The epsilon amino groups on the exposed lysine residues are available for chemical conjugation to any given protein, and can thus act as antigen carriers. The availability of amino groups on the surfaces of TEV particles was determined and the immune response to TEV evaluated.

**Results:**

Using a biotin-tagged molecule that reacts specifically with amino groups, we found that the TEV capsid protein has amino groups on its surface available for coupling to other molecules *via *crosslinkers. Intraperitoneal TEV was administered to female BALB/c mice, and both their humoral and cellular responses measured. Different IgG isotypes, particularly IgG2a, directed against TEV were induced. In a cell proliferation assay, only spleen cells from vaccinated mice that were stimulated *in vitro *with TEV showed significant proliferation of CD3^+^/CD4^+ ^and CD3^+^/CD8^+ ^subpopulations and secreted significant amounts of interferon γ.

**Conclusions:**

TEV has surface amino groups that are available for chemical coupling. TEV induces both humoral and cellular responses when administered alone intraperitoneally to mice. Therefore, TEV should be evaluated as a vaccine adjuvant when chemically coupled to antigens of choice.

## Background

Tobacco etch virus (TEV) belongs to the genus *Potyvirus*, the largest and economically most important genus of the recognized plant virus groups and families [1]. The genomes of the potyviruses are single positive-stranded RNAs, surrounded by approximately 2,000 subunits of the coat protein (CP) [2]. A previous study has demonstrated that the CP amino and carboxy termini of several potyviruses are located on the surface of the infectious particle and bear the most immunogenic epitopes [3]. Based on biochemical and immunological evidence, two other studies have suggested that the first 29 amino acids of the TEV capsid protein are hydrophilic and are located at or near the particle's surface [4,5].

Generally, viruses induce good immune responses, which are dependent on their surface structures. These structures consist of one or a few proteins and are highly organized and repetitive in nature. This repetitiveness could be recognized by the immune system as a pathogen-associated geometric pattern similar to pathogen-associated molecular patterns [6]. Viruses are good immunogens because they facilitate the crosslinking of B-cell receptors, enhancing the host antibody response [7,8]. Viruses are also efficiently internalized, processed, and presented by antigen-presenting cells [9]. These features make viruses good candidates for the presentation of foreign antigens on their surfaces. By exploiting these features, several plant viruses have been used as antigen-presenting platforms for the development of subunit vaccines directed against a variety of human and animal pathogens. This is normally achieved by inserting DNA sequences in-frame with the CP-encoding gene. The viruses used for this purpose include the tobacco mosaic virus (TMV) [10,11], cowpea mosaic virus [12-15], cucumber mosaic virus (CMV) [16], alfalfa mosaic virus [17], potato virus × [18], and papaya mosaic virus (PapMV) [19]. Until now, only one potyvirus, plum pox virus, has been used as a platform for displaying foreign amino-acid sequences on its surface [20,21].

One limitation of the translational fusion approach is the size of the sequence that can be inserted without compromising the capsid protein self-assembly, which is fundamental to stimulating a good immune response. Generally, this size cannot exceed 20 amino acids, although larger sequences should be exposed [22]. One alternative to translational fusions is coupling the viruses to peptides or complete antigens through chemical crosslinkers that bind specifically to groups present in the side chains of some amino acids. With this strategy, several plant viruses have been used for the surface display of exogenous proteins. In the cowpea mosaic virus, an icosahedral virus model that has been genetically modified for accurate chemical conjugation, 100% occupancy of CP monomers by complex molecules was demonstrated, with the retention of the biological activity of the attached proteins [23]. Another study has shown that TMV is an effective vaccine carrier for stimulating peptide-specific immunity to both single and multivalent vaccines [24]. The presentation of whole protein on TMV has also been demonstrated, expanding the utility of TMV as a vaccine scaffold by the genetic manipulation of both TMV and the presented antigen [25]. There is apparently no limitation on the antigen size with this approach and a variety of epitopes can be exposed on a single viral particle. However, this assumption must be evaluated for each specific case.

When we analyzed several reported CP sequences from TEV, we realized that the TEV CP amino terminus is rich in positively charged residues, predominantly lysines. Lysine residues are often utilized for chemical coupling *via *their epsilon amino groups. If these lysine residues were exposed on the viral surface, they would be available for chemical conjugation with a variety of antigens. In this study, we demonstrated that TEV CP lysines exposed on the particle surface can be used for antigen coupling through chemical conjugation. We also evaluated the immune response to the virus in a mouse model. Based on these findings, we propose that TEV be evaluated as an adjuvant for subunit vaccines.

## Results and discussion

### Why TEV is a good candidate vaccine adjuvant

We consider that TEV offers several advantages as a carrier for antigen presentation. There has been no report that TEV or any other plant virus replicates effectively in humans or animals. Moreover, yields of TEV from infected tobacco plants are high and its purification is relatively easy, providing enough material for large-scale formulations. Most importantly, by substituting a few amino acids in the CP and the helper-component proteinase, nonaphid-transmissible mutants can be generated to prevent the virus spreading [26,27].

### TEV CP *in silico *model

**Figure 1:**
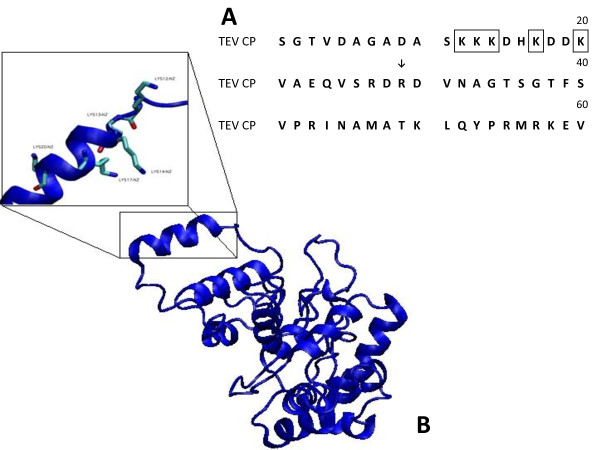
**Partial amino acid sequence and structural model of TEV capsid protein**. **A**) Predicted sequence for the first 60 amino acids of TEV-NAY capsid protein (CP). Solid arrow indicates the first 29 amino acids of CP, located on the virion's surface. Solid boxes indicate lysines present in the surface-exposed region of CP that are available for chemical conjugation. **B**) Predicted structure for TEV-NAY CP, with the solvent-accessible N-terminal lysine-rich stretch. A closer view of localized lysines (K12, K13, K14, K17, and K20) is shown in the rectangle.

The TEV used in this study, designated TEV-NAY, was a field isolate from Nayarit, Mexico. The TEV-NAY isolate was propagated and purified as described in the Methods section and its genetic material was isolated. Its CP cistron was amplified, cloned, and sequenced. As shown in Figure 1A, the TEV CP amino region, spanning the first 29 amino acids, contains approximately five lysines in nine residues [4,5]. Lysine reacts well with N-hydroxy succinimide (NHS) esters without the assistance of neighboring amino acids [28], and NHS esters are used extensively for chemical crosslinking.

From the various sequences of several *Potyvirus *CPs, we generated *ab initio *models using the Rosetta software (http://www.pyrosetta.org/) and selected the sequence that most broadly represented the biochemical features previously reported [29,30]. The best model was used as the template to generate the homologous model (http://salilab.org/modeller/) of the TEV-NAY CP sequence. This model provides valuable structural information that strongly suggests the availability of the epsilon amino groups of surface-exposed lysine 12 (K12), K13, K14, K17, and K20 for chemical conjugation to a variety of antigens (Figure 1B).

### TEV has amino groups exposed on its surface

**Figure 2:**
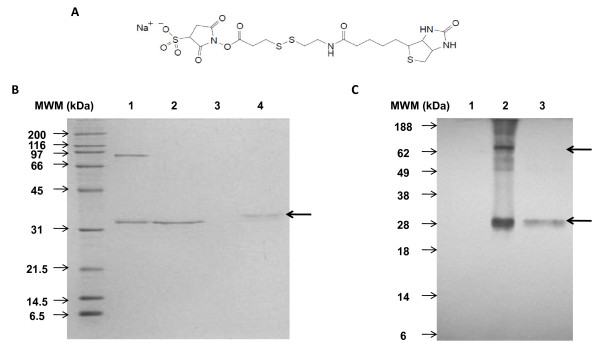
**Availability of surface-exposed amino groups on TEV capsid protein for chemical conjugation to biotin-tagged reagent**. **A**) Chemical structure of biotin-tagged reagent, Sulfo-NHS-SS-Biotin: the NHS ester group reacts specifically with the amino groups of amino acids; the spacer arm contains an S-S bond that can be cleaved by treatment with the reducing agent 2-mercaptoethanol; and biotin is attached on the other side of the spacer arm. **B**) 12% SDS-PAGE of untreated virus under nonreducing and reducing conditions (lanes 1 and 2, respectively) and biotinylated virus under nonreducing and reducing conditions (lanes 3 and 4, respectively) stained with Coomassie Blue. **C**) Western blot of treated and untreated samples. Lane 1: untreated virus under reducing conditions; lanes 2 and 3, biotinylated virus under nonreducing and reducing conditions, respectively. A streptavidin-horseradish peroxidase conjugate was used as the detection reagent. MWM, molecular weight marker. Bold arrows indicate main biotinylation products.

Based on the observations described above, we indirectly tested whether the virions from the TEV-NAY isolate carried exposed amino groups on their surfaces. First, the integrity of the pure TEV virions was confirmed by electron microscopy (data not shown). Second, the availability of the amino groups on the TEV particles was evaluated with Sulfo-NHS-SS-Biotin. This reagent is composed of NHS, which covalently binds the amino groups exposed on the surface of any protein, attached to biotin *via *a spacer arm. The biotin can be removed from the reagent with a reducing agent (Figure 2A). After TEV was incubated with Sulfo-NHS-SS-Biotin, the sample was subjected to sodium dodecyl sulfate polyacrylamide gel electrophoresis (SDS-PAGE) under reducing or nonreducing conditions and stained with Coomassie Blue or transferred to a membrane for western blot analysis to verify biotin binding. Coomassie Blue staining revealed a band slightly larger than the band present in the untreated TEV sample (Figure 2B), which we presumed corresponded to TEV CP, which has a calculated mass of 32 kDa [31]. The observed increase in mass suggests the coupling of TEV CP with the reagent, because one molecule of Sulfo-NHS-SS-Biotin attached to the 32 kDa TEV CP represents only a 606.9 Da increase in molecular weight. This increase results in a discernible shift on gel electrophoresis, allowing us to assess the extent of TEV CP conjugation. Because no unconjugated TEV CP band was observed after the Sulfo-NHS-SS-Biotin treatment, we assumed that 100% occupancy was achieved after incubation with a 20-fold molar excess of Sulfo-NHS-SS-Biotin for 1 h at room temperature. This contrasts with a study of a modified TMV [25], in which a 240-fold molar excess of NHS-PEO_4_-biotin was required to obtain fully biotinylated TMV. To confirm that Sulfo-NHS-SS-Biotin was indeed bound to the TEV after treatment, a western blot analysis was performed using horseradish peroxidase (HRP)-conjugated streptavidin as the conjugate (Figure 2C). Under nonreducing conditions, the main band detected corresponded to TEV CP, but a 90 kDa band was also clearly observed, which was also present under nonreducing conditions after Coomassie Blue staining. This larger band might correspond to either different oligomeric states of CP after SDS treatment or to an association between the terminal CPs and either helper component protein, viral genome-linked protein, or cytoplasmic inclusion protein, which are thought to be present at one end of CP in a subpopulation of virions [32-34]. Under reducing conditions, the TEV CP signal was considerably reduced because of the partial separation of biotin from Sulfo-NHS-SS-Biotin. An interesting observation was the presence of a second band smaller than the major conjugation product after gel electrophoresis and western blotting, which could indicate the coupling of more than one biotin to a single TEV CP. We expected the TEV CP signal to disappear completely under reducing conditions, and this may have been achieved by incubating the sample with reducing agent for a longer period of time or by using dithiothreitol rather than 2-mercaptoethanol. Another interesting observation was that there was no signal for the 90 kDa form of CP under reducing conditions. In summary, our results strongly suggest that TEV particles are amenable to chemical coupling through their surface-exposed amino groups.

### TEV induces antibody production

**Figure 3:**
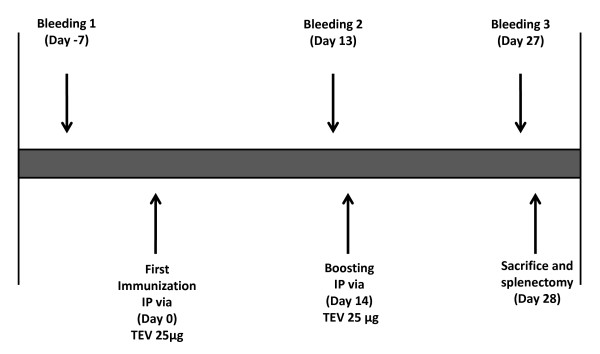
**Immunization and serum collection schedule**. To evaluate the TEV-associated immune response in a mouse model, two groups of five female Balb/c mice were tested, designated C and TEV. Pure TEV (25 μg) or an equal volume of 20 mM Tris (pH 8.0) were administered to the TEV or C group, respectively. The mice were immunized intraperitoneally (IP) on day 0 and boosted on day 14. During the experiment, the mice were bled three times, on days -7 (B1), 13 (B2), and 27 (B3). The animals were killed at the end of the experiment on day 28, before splenectomy.

**Figure 4:**
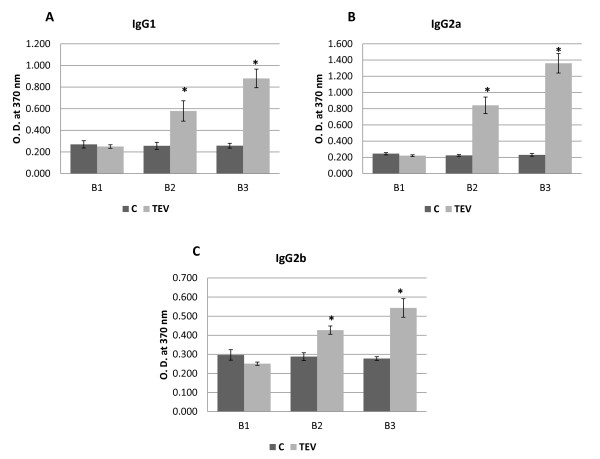
**Analysis of the antibody responses with ELISA**. Serum samples were collected from each group (C and TEV) at different times (see the immunization and bleeding schedule in Figure 3), diluted 1:40 with PBS, and tested for anti-TEV IgG1 (**A**), IgG2a (**B**), and IgG2b (**C**) antibody isotypes. Aliquots (5 μg per well) of TEV were fixed to the plate. Readings were performed with the TMB colorimetric substrate at 370 nm until reaction saturation. The results are expressed as the means ± standard deviations (SD) of the corresponding measurements. Statistically significant differences are indicated with an asterisk (*P < 0.05).

We next immunized female BALB/c mice (Figure 3) to evaluate the immune response induced by TEV in this model. Mice were injected with 25 μg of TEV or viral diluent (20 mM Tris, pH 8.0) on days 1 and 14 and were bled on days -7, 13, and 27. Their sera were analyzed for the relative levels of anti-TEV antibodies. Three immunoglobulin G (IgG) isotypes against TEV were measured: IgG1, IgG2a, and IgG2b. The isotype with the highest titer was IgG2a, followed by IgG1 and IgG2b (Figure 4). Our results are similar to those obtained with the intraperitoneal immunization of mice with PapMV, a potexvirus that, like TEV, is a flexible rod, but it is 1.5 times wider and two times shorter than TEV [35]. In another report, very similar results were observed when PapMV-virus-like particles harboring a hepatitis C virus epitope were administered subcutaneously, but the response evaluated in that study was against the hepatitis C virus epitope [36]. Another study reported that TMV, chemically decorated with either green fluorescent protein (GFP) or the canine oral papillomavirus L2 antigen, induced antigen-specific antibodies in mice and guinea pigs immunized with low doses of TMV complex without adjuvant [25]. Our results show that TEV is a strongly immunogenic particle, and can induce an antibody response in which the IgG2a subclass is the major antibody product, a bias that can be indicative of a stronger Th1 immune response.

### TEV induces T-lymphocyte proliferation

**Figure 5:**
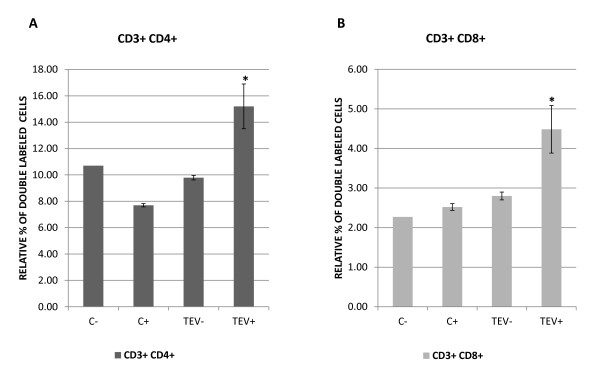
**In vitro proliferation assay of T-cell subpopulations derived from spleen cells of TEV-vaccinated mice**. Mononuclear cells were isolated from the spleens of TEV-immunized (TEV) and nonimmunized (C) mice. To evaluate lymphocyte proliferation, the cultured cells of both groups were either unstimulated (-) or stimulated with 5 μg of virus (+). The collected cells from each treatment were triple-stained with fluorescent antibodies (PE-Cy5-labeled anti-CD3, FITC-labeled anti-CD4, and PE-labeled anti-CD8) and analyzed in a flow cytometer. The graphs show the relative percentages of double-stained cells, CD3^+^/CD4^+ ^for helper T cells (A) and CD3^+^/CD8^+ ^for cytotoxic T cells (B). The results are expressed as the means ± standard deviations (SD) of the corresponding measurements. Statistically significant differences are indicated with an asterisk (*P < 0.05).

To examine the capacity of TEV to induce a T-lymphocyte response, immunized and control mice were killed 28 days after their first immunization, and the mononuclear cells were isolated from their spleens with a Ficoll-Paque density gradient. The isolated cells were then grown in medium and either stimulated or not stimulated with TEV for three days. After this incubation period, the cells were harvested and the CD3^+^/CD4^+ ^(T-helper lymphocyte) and CD3^+^/CD8^+ ^(T-cytotoxic lymphocyte) subpopulations were analyzed by flow cytometry. Only cell cultures from the immunized mice that were further stimulated *in vitro *with TEV showed a significant increase in both subpopulations (Figure 5). This indicates that a memory of TEV was established in both T-helper and T-cytotoxic lymphocytes. A similar study hypothesized that CD4^+ ^T cells improved the efficacy of TMV-antigen complex and thus inhibited tumorigenesis [24]. It has also been observed that the formation of a complex between TMV and a cargo molecule is required for the robust induction of activated antigen-specific CD8^+ ^T cells [25].

### TEV induces interferon γ (IFNγ) secretion in cultured splenocytes

**Figure 6:**
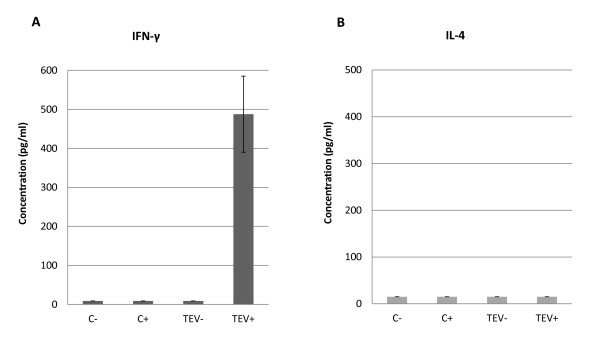
**IFNγ and IL4 levels in stimulated splenocytes from mice immunized with TEV**. IFNγ and IL4 levels in the supernatants of cultured splenocytes from immunized or nonimmunized mice following restimulation with TEV or no treatment were measured by ELISA. The graphs show the IFNγ (A) and IL4 concentrations (B) in picograms per milliliter (pg/mL) for each treatment: nonimmunized/unstimulated (C-), nonimmunized/stimulated (C+), immunized/unstimulated (TEV-), and immunized/stimulated (TEV+). The cytokine levels were calculated using standard curves generated with known concentrations of recombinant proteins. The data are presented as the mean cytokine concentrations ± SD of triplicate measurements. "Undetectable" (§) means below the lowest point on the standard curve.

To determine whether TEV preferentially induces a Th1 or Th2 response, two cytokines, IFNγ and interleukin 4 (IL4), were measured in the supernatants of cultured splenocytes. IFNγ and IL4 are mediators of the Th1 and Th2 responses, respectively. The results, shown in Figure 6, indicate that TEV induces a bias towards the Th1 response, because TEV treatment caused IFNγ secretion. It is noteworthy that this response was observed only in the splenocytes from TEV-vaccinated animals that were further stimulated with TEV. No levels of IL4 were detected after any treatment. A previous study of CMV, an icosahedral virus, showed that CMV itself induced IFNγ secretion in peripheral blood mononuclear cells that had not been primed against the virus, indicating that CMV induces a dominant Th1 immune response [37]. Furthermore, a bivalent formulation of TMV conjugated to toxin-derived peptides, T-helper epitopes, or peptides for enhanced antigen uptake, significantly improved the immune responses in mice, as measured by the levels of IFNγ-secreting cells, and their survival after lethal challenge with tumor cells without adjuvant [24]. The results of the present study indicate that TEV, which induces the secretion of a molecule that mediates the Th1 response, could be used as a vaccine adjuvant when a Th1 response is fundamental to the generation of protective immunity.

## Conclusions

The following conclusions can be drawn from the results presented here. First, the amino groups of CP lysines are exposed on the surfaces of infectious TEV particles, as revealed by *in silico *modeling and biotinylation experiments. This is a remarkable property that may be unique to TEV. Such amino groups are probably available for chemical coupling to antigens of choice. Therefore, no modification of the native TEV CP is necessary because the amino-acid side chains commonly targeted for conjugation are naturally present in TEV field isolates. Whether these groups can be coupled to large antigens will primarily depend on the spacer arm length present in the crosslinker utilized for this purpose and the mass and tertiary structure of the antigen, and is yet to be determined. We expect no change in the virus structure after biotin coupling, although we did not explicitly assess this in this study. In previous work with a modified TMV, the complete biotinylation of the CP did not alter the size distribution of the virus. Nonetheless, when biotinylated TMV was loaded with green fluorescent protein-streptavidin at 26% capacity, a notable reduction in rod length was documented, leading to the conclusion that the load capacity of the virus depends on the size of the antigen [25]. Second, TEV induces T-cell and antibody responses when administered alone to mice. Third, TEV induces IFNγ secretion, which is a mediator of the Th1 response. Therefore, TEV could be a useful adjuvant against some intracellular pathogens where this type of response is critical for adequate protection. Further experiments are required to determine whether TEV can induce type-I interferon production in immune cells through the interaction of its single-stranded RNA with Toll-like receptor 7 [38]. In summary, we propose a thorough evaluation of TEV as a vaccine adjuvant, to increase the density of the potentially displayed antigens.

## Methods

### Virus preparation

The virus used in this study was a field isolate from Nayarit, Mexico, designated TEV-NAY, and was propagated in about 20 Burely B49 *Nicotiana tabacum *plants. When the symptoms of infection were fully expressed systemically, 200 g of leaf tissue was ground in a blender with two volumes of 20 M HEPES (pH 7.5), butanol (18% final concentration), and sodium sulfite (0.1% final concentration) for 2 min. The glass-fiber-filtered extract was centrifuged for 5 min at 1500 × *g*. The first precipitation was performed with PEG 8000 (4% w/v), Triton X-100 (1% w/v), and NaCl (0.1 M final concentration), after stirring for 1 h at 4°C and centrifugation at 3000 × *g*. Using a glass homogenizer, the pellet was resuspended in 20 mM HEPES (pH 7.5) in one quarter of the original volume. The second precipitation was performed with 8% PEG 8000 and no Triton X-100. The pellet was resuspended in 3 mL of the same HEPES buffer and placed on 3.5 mL of CsCl for a final centrifugation at 153,400 × *g *at 4°C for 10-12 h. The viral band was dialyzed against 0.01 M HEPES overnight at 4°C. A yield of up to 18 mg per 100 g of infected tissue was usually obtained. Viral integrity and purity were verified with electron microscopy.

### Nucleic acid purification, amplification, and sequencing

Total RNA from TEV-infected tobacco plants was used as the template to amplify the viral CP cistron, in an RT-PCR reaction. The primers were designed according to the available TEV sequences from NCBI GenBank, directed towards the two sets of five amino acids flanking the viral CP cistron. The amplified product was cloned in the pGEM-T Easy vector (Promega, USA) and sequenced with an ABI Prism Sequencer (Applied Biosystems, USA). The nucleotide sequence was compared with the sequences available at NCBI to confirm its identity.

### *In silico *modeling of TEV CP

Several potyviruses (papaya ringspot virus, sugarcane mosaic virus, bean common mosaic virus, bean common mosaic necrosis virus and TEV) were used to generate 50,000 models per sequence using the PyRosetta software, with the 5 Å grouping restriction of the root mean square deviation between the Cαs, and selecting the model most representative of the largest group. The lateral chains were added, refined, and relaxed later on the model. Validation and ranking with the ConSurf and ConQuass software, respectively, were then performed. The final selection was made with consideration of previously reported biochemical data, the secondary structures predicted by PSIpred, and the model ranking by Procheck. This final model was used as the template to generate homologous models of the TEV-NAY CP sequence using Modeller.

### TEV surface amino group availability assay

Using a biotin-tagged reagent, Sulfo-NHS-SS-Biotin (Thermo Scientific Pierce, USA), an experiment was performed to determine the presence of amino groups exposed on the surface of TEV particles, following the manufacturer's instructions. Briefly, TEV was dialyzed overnight against 2,000 volumes of phosphate-buffered saline (PBS; pH 7.2) at 4°C, using Slide-A-Lyzer Dialysis Cassette Kit for 0.5-3.0 mL samples (Thermo Scientific Pierce). The dialyzed TEV was then mixed with a 20-fold molar excess of Sulfo-NHS-SS-Biotin prepared from a fresh 10 mM Sulfo-NHS-SS-Biotin solution, and incubated for 1 h at room temperature. The sample was then immediately loaded onto a gel for SDS-PAGE under nonreducing or reducing conditions (5% 2-mercaptoethanol) and stained with Coomassie Blue. A portion of the sample was subjected to SDS-PAGE as described above, and blotted onto Hybond ECL nitrocellulose membrane (GE Healthcare, USA). The blot was incubated with HRP-labeled streptavidin (KPL, USA) and then visualized with HRP Color Development Reagent (BioRad, USA), as recommended by the manufacturer.

### Mouse immunization and bleeding

Ten four-week-old female BALB/c mice (Harlan, Mexico) were randomly divided into two groups of five animals each, and maintained throughout the experimentation period in the Vaccine Evaluation Module-Animal Experimentation Laboratory, Centro de Investigación y Asistencia en Tecnología y Diseño del Estado de Jalisco. The animals were cared for according to good laboratory practice guidelines. Before the experiments, the animals were allowed to adapt to these conditions for one week.

After the adaptation period, the mice were bled from the tail and individual preimmune sera were collected. One week later, the mice were immunized intraperitoneally. The immunization scheme is shown in Figure 3. On day 0, the first immunization (priming) was performed; one group, designated TEV, was inoculated with 25 μg of virus solution, and the other group, used as the control and designated C, was inoculated with the same volume of buffer solution (20 mM Tris, pH 8.0). The mice were bled a second time 13 days after priming. A second identical immunization (booster) was performed on day 14. The mice were bled a third time 27 days after priming. The individual serum samples were stored at -70°C until analysis.

### Determination of antibody titers with enzyme-linked immunosorbent assays (ELISAs) of sera

The sera obtained from the blood samples collected in the immunization experiments were used to determine the relative levels of mouse IgG1, IgG2a, and IgGb isotypes, using a mouse monoclonal antibody isotyping kit (Sigma, USA), following the manufacturer's procedure for antigen-mediated ELISA, with slight modifications. Briefly, 5 μg of TEV diluted in coating buffer (100 mM carbonate buffer, pH 9.6) was added to each well of a 96-well MaxiSorp Immuno Plate (NUNC, USA) and incubated at 4°C overnight. The plate was then blocked with 5% skim milk in PBS-0.05% Tween 20 for 2 h at room temperature. Individual serum samples were diluted 1:40 in PBS and loaded into the wells in duplicate and the plate was incubated for 1 h at room temperature. Anti-mouse-isotype antibody diluted 1:1000 in PBS was then added and the samples incubated for 1 h. The plate was then incubated with 1:5000 HRP-conjugated anti-goat IgG antibody (Sigma, USA). Finally, TMB liquid substrate (Sigma, USA) was added for color development, which was monitored in an xMark Microplate Absorbance Spectrophotometer (Bio-Rad, USA) at 370 nm every 5 min until reaction saturation.

### Isolation and purification of splenic lymphocytes

All the animals were killed on day 28, and their spleens were collected under sterile conditions inside a laminar flow hood, washed several times with PBS (pH 7.4), and maintained in RPMI-1640 medium (Gibco, USA) supplemented with 10% fetal bovine serum (Gibco, USA), penicillin-streptomycin-neomycin antibiotic mixture (Gibco, USA), and GlutaMAX (Gibco, USA) until homogenization. The spleens were homogenized by hand in a PYREX homogenizer and the cell suspensions were passed through a 40 μm cell strainer (BD Falcon, USA) to eliminate cellular and tissue debris. The filtered homogenates from either the TEV or C treatments were pooled and the suspensions obtained were loaded onto a density gradient of 1.077 g/mL Ficoll-Paque PLUS (GE Healthcare, USA) in a 1:1 ratio and centrifuged at 2,200 × *g *for 25 min at room temperature. The mononuclear-cell-enriched fraction was collected, and washed once with RPMI-1640 medium. The cells were counted in a Neubauer chamber with 0.4% Trypan Blue Stain solution (Gibco, USA).

### T-cell proliferation assay

Each pool of cells (TEV or C) was dispensed into six wells of a flat-bottom 24-well cell culture dish (Costar, USA) to a final concentration of 5 × 10^6 ^cell/500 μL of RPMI-1640 medium. Three wells were stimulated with 5 μg of TEV, and the other three wells were not stimulated. The plate was incubated at 37°C for 72 h under 5% CO_2_. After 24 h, 500 μL of fresh RPMI-1640 medium was added to each well. After 48 h, 200 μL of the supernatant was collected from each well for cytokine profiling (below) and replaced with 200 μL of fresh medium. At 72 h, the cells were collected by centrifugation at 250 × *g *for 5 min, washed once with PBS, and immediately stained for flow cytometry.

To identify the T-cell subpopulations in the splenic cell cultures, a set of three different antibodies was used: phycoerythrin (PE)-Cy5-labeled anti-mouse CD3ε (BioLegend, USA), fluorescein isothiocyanate (FITC)-labeled anti-mouse CD4 (BioLegend, USA), and PE-labeled anti-mouse CD8a (BioLegend, USA). Cultured splenic cell replicates were pooled, divided into aliquots (10^6 ^cells in a volume of 100 μL) and triple-labeled following the manufacturer's instructions. The cells were then washed once with PBS, fixed in an appropriate volume of 0.05% paraformaldehyde in PBS, and stored at 4°C until analysis in triplicate in a Beckman Coulter EPICS XL-MCL Flow Cytometer. A control unstimulated sample from nonvaccinated mice with no fluorescent marker or with a single fluorescent marker was used to identify the cell population or to adjust the color compensation settings for the multicolor analysis, respectively. All data were analyzed with the Beckman Coulter System II software.

### Determination of IFNγ and IL4 levels in the culture supernatants

This assay was conducted to identify any Th1/Th2 bias in response to TEV, based on the expression of the IFNγ and IL4 cytokines. For this purpose, Quantikine Mouse IFN-γ and Mouse IL-4 ELISA Kits (R&D Systems, USA) were used to analyze the supernatants from the T-cell-proliferation assay cultures, according to the instructions of the manufacturer. The supernatants (200 μL) of the cell cultures were collected after 48 h, centrifuged at 3,000 × *g *for 5 min, and stored at -70°C until analysis. The cytokine levels were determined using standard curves generated with known concentrations of the recombinant proteins. The results are expressed in picograms per milliliter (pg/mL).

### Statistical analysis

Differences between groups were analyzed with Duncan's test. Probability values (*P *values) less than 0.05 were considered to be significant. Excel and StatGraphics were used for the statistical analysis.

## Competing interests

The authors declare that they have no competing interests.

## Authors' contributions

CAMC bled the mice, performed the conjugation experiment, antibody titering, and cytokine profiling, and also helped to draft the manuscript. AMA performed the mouse splenectomies and established and maintained the spleen cell cultures. RHG immunized the mice. PCOL performed and interpreted the flow cytometry. GCC and MCT established the *in silico *TEV CP model. LSR propagated and purified the TEV and helped to draft the manuscript. AGO conceived the study, participated in its design and coordination, and drafted the manuscript. All the authors have read and approved the final manuscript.
